# Parental Risk for Suicide and Attachment Patterns Among Adolescents With Borderline Personality Disorder. A Clinical-Based Study

**DOI:** 10.3389/fpsyt.2018.00771

**Published:** 2019-01-11

**Authors:** Ana Moscoso, Mario Speranza, Veronique Delvenne, Maurice Corcos, Alexandra Pham-Scottez

**Affiliations:** ^1^Service Universitaire de Psychiatrie de l'Enfant et de l'Adolescent, Centre Hospitalier de Versailles, Versailles, France; ^2^“Recherches Cliniques et en Santé Publique sur les Handicaps Psychique, Cognitif et Moteur” (HANDIReSP), Université de Versailles Saint Quentin en Yvelines, Versailles, France; ^3^Service de Pédopsychiatrie, Hôpital Universitaire des Enfants Reine Fabiola, Brussels, Belgium; ^4^Département de Psychiatrie de l'Adolescent et du Jeune Adulte, Institut Mutualiste Montsouris, Paris, France; ^5^CMME, Hôpital Sainte Anne, Paris, France

**Keywords:** borderline personality disorder, BPD, parental suicidal attempts, preoccupied attachment, adolescent

## Abstract

**Introduction:** Experiencing adverse life events and early disturbed patterns of interaction are crucial determinants for the development of Borderline Personality Disorder (BPD). Parental suicidal attempts can be considered a major adverse life event and a potentially traumatic experience. The aim of this study was to examine differences in parental suicidal attempts among a BPD adolescent population vs. a matched control group. We also aimed to understand if attachment styles and the number of parental suicidal attempts predicted the severity of borderline symptomatology.

**Methods:** Our study (EURNET BPD) comprised 85 BPD adolescents and 85 matched controls. Axis II disorders were investigated using the French version of SIDP-IV. Parental suicidal behaviors were assessed during a face-to-face interview with the adolescent. Attachment style was assessed with the Relationship Questionnaire (RQ).

**Results:** Parents of BPD adolescents made more suicidal attempts than controls (34 vs. 11%; chi2 = 13.8, *p* < 0.001). The linear regression showed that the best model explaining the severity of borderline symptomatology (*R*^2^ adjusted = 0.15, *F* = 4.65, *p* < 0.001) was a model including the number of suicidal attempts realized by the parent (standardized beta = 0.28, *p* = 0.009) and the preoccupied attachment style of the adolescents toward their parents (standardized beta = 0.21, *p* = 0.043).

**Conclusion:** Our results highlight the usefulness of assessing parental suicidal behavior when interviewing patients with BPD traits. This could be useful in clinical practice as an early clue to identify patients with potentially severe clinical profiles and parents needing a specific support. Further research is needed to confirm our findings.

## Introduction

Borderline Personality Disorder (BPD) in adolescence is a severe mental disorder characterized by pervasive and persistent patterns of instability—affective, relational, of the self—and impulsivity. The condition is associated with frequent risk-taking and self-harm behaviors and many psychiatric comorbidities as well as severe psychosocial impairments, and extensive use of mental health services. Cumulative prevalence rates suggest that 1.4% of young people will meet diagnostic criteria for BPD by age 16 years, rising to 3.2% by age 22 years (1), but more importantly BPD is common among adolescents within mental health settings, with an estimated prevalence of 11% in psychiatric outpatients and up to 50% in inpatient settings (2).

Although currently there isn't a unifying model to describe and understand in depth the complex phenomenon of borderline personality, genetics, and environmental factors are both thought to play a role. Concerning environmental factors, experiencing adverse life events and trauma have been largely investigated in the past (3, 4). However, not all individuals who suffer maltreatment will develop a BPD (5). Early disturbed patterns of interaction constitute a risk factor for emotional vulnerability and are crucial determinants for the development of BPD. Maladaptive parenting, including parental hostility, overprotective and rejecting parenting styles and attachment disorganization emerged as strong predictors of BPD symptoms (6, 7). The quality of primary relationships is so important that it might modulate in one way or the other the integration of early traumatic events (5) and therefore the development of personality.

To our knowledge, parental suicide hasn't been studied specifically in adolescents with BPD. Nevertheless, it has been recognized that parental suicide attempts can have a major impact in the offspring and can be considered a major adverse life event and a potentially traumatic experience (8). Parental suicidal attempt can also question the attachment in the young person, through the fear of potential abandonment (9). Besides, behind a suicidal attempt, psychopathology and social adversity is often hidden (10).

The primary aim of this study was to examine differences in parental suicidal attempts among a BPD population vs. a matched control group. We also aimed to test whether attachment styles and the number of parental suicidal attempts predicted the severity of borderline symptomatology.

## Methods

### Participants

The study sample was drawn from a European research project investigating the phenomenology of BPD in adolescence [the European Research Network on Borderline Personality Disorder, EURNET BPD; see (11) for a full description of the study methodology]. The research network was composed of five specialist psychiatric centers for adolescents and young adults in France, Belgium, and Switzerland. The final study population comprised 85 BPD adolescents (11 boys, 13%, and 74 girls, 87%). The mean age was 16.5 years (SD = 1.4). 67% (*N* = 57) were inpatients. The control sample included 85 healthy adolescents individually matched for gender, age and socio-economic status. Control subjects were excluded if they had a history of or ongoing psychiatric follow-up, and if they were positive for a DSM-IV diagnosis of personality disorder. The study was approved by our local institutional review board and a written informed consent was obtained from the adolescents and at least one of their parents.

### Assessments

All subjects completed a research protocol (consisting of a diagnostic evaluation of Axis I and Axis II disorders) and a self-administered questionnaire to collect socio-demographic and psychopathological data. Axis II disorders were investigated using the French version of SIDP-IV, which is known to have good psychometric properties in adolescents and young adults (12). Borderline severity for each of the 9 criteria was coded as absent (0), subliminal (1), present (2), and severe (3). Borderline severity scores thus varied from 0 to 27. The inter-rater reliability for SIDP-IV was calculated from independent ratings of 10 videotaped interviews. The Kappa coefficient for agreement on the presence or absence of a BPD was very high (0.84) and the values for the presence/absence of other personality disorders ranged from 0.54 to 1.

Parental suicidal behaviors (presence/absence; number of suicidal attempts) were assessed during the face to face interview with the adolescent. The age of the adolescent at the time of the first suicidal attempt of the parent was also recorded.

Attachment was assessed with the Relationship Questionnaire (RQ) (13), a valid and reliable self-report measure of adult attachment [(14, 15) measuring strength of attachment along two dimensions of anxiety and avoidance. The RQ generates a negative to positive score (−12 to +12) on the attachment dimensions of anxiety and avoidance, with higher scores indicating less anxiety and avoidance respectively.

### Statistical Analysis

Categorical variables (socio-demographics, presence/absence of parental suicidal attempts, insecure/insecure attachment) were compared between groups (borderline and control sample) using chi-square analyses. To compare the mean scores of the borderline and the control sample on the number of parental suicidal attempts and on attachment styles we used a one-way anova. Bivariate associations between attachment styles and the overall severity of borderline symptomatology were examined with Pearson correlations in the borderline sample only. Finally, to test whether attachment styles and the number of parental suicidal attempts predicted the severity of borderline symptomatology we conducted a linear regression analysis with a stepwise procedure. All analyses were conducted using SPSS (25th version).

## Results

### Parental Suicidal Attempt

34% (*N* = 29) adolescents from the borderline group reported a suicidal attempt made by their parents compared to 11% (N=9) from the control sample. This difference was statistically significant (chi2 = 13.8, *p* < 0.001) (Table 1). All the parents of the control group had made only one suicidal attempt, while the parents of the borderline group reported several suicidal attempts (4.2, SD 3.7) (*F* = 5.7, *p* = 0.022) with some parents reporting up to 10 suicidal attempts. Mothers accounted for the majority of the suicidal attempts (73%). Borderline adolescents reported that the first suicidal attempt of their parents happened during their childhood and early adolescence (mean 12.7, SD 4.0).

**Table 1:** Comparison between the borderline and control groups regarding parental suicidal attempts and attachment patterns.

**Variables**	**Borderline sample (*N* = 85)**	**Control sample (*N* = 84)**	**Statistics**	
	**M ± SD/(%)**	**M ± SD/(%)**	**F/Chi2**	***P***
Age	16.5 ± 1.4	16.2 ± 1.3	F = 2.71	0.10
Sex (men) (%)	11 (13%)	20 (24%)	F = 3.3	0.70
Parental suicidal attempt (present/absent) (%)	29 (24%)	9 (11%)	Chi2 = 13.8	**0.001**
N° of parental suicidal attempts	4.2 ± 3.7	1 ± 0	F = 5.7	**0.001**
RQ Secure attachment	2.9 ± 1.6	4.2 ± 1.6	F = 23.4	**0.001**
RQ Fearful attachment	4.0 ± 1.9	2.7 ± 1.5	F = 19.3	**0.001**
RQ Preoccupied attachment	4.2 ± 2.0	3.1 ± 1.7	F = 12.0	**0.001**
RQ Dismissing attachment	2.5 ± 1.8	2.5 ± 1.5	F = 0.01	0.9
Secure/insecure attachment (%)	34%	23%	F = 25.1	**0.001**

*Statistically significant differences appear in bold*.

### Attachment Styles

Data from the RQ showed that borderline adolescents showed more insecure attachments than controls (34.5 vs. 23%, *F* = 25.1, *p* = 0.001) (Table 1). Borderline adolescents had significantly lower scores on secure attachment and significantly higher scores on the fearful and preoccupied styles of attachment.

### Correlations to Borderline Severity

Pearson correlations showed that the number of parental suicidal attempts (*r* = 0.37, *p* < 0.05) was significantly correlated to the severity of borderline symptomatology, with fearful and preoccupied attachment styles showing a trend to a significant correlation (Table 2).

**Table 2:** Multiple regression model predicting the severity of borderline symptomatology.

**Predictive variables**	**Estimate**	**SE**	***b***	***T***	***P***
N° of parental suicidal attempts	3.79	1.4	0.28	2.68	0.009
Preoccupied attachment	0.50	0.2	0.21	2.05	0.043

*Linear regression model. Stepwise method. Dependent variable: Severity of borderline symptomatology (SIDP-IV), Independent variables: attachment styles (RQ), n° of parental suicidal attempts, n° of adolescent suicidal attempts. Sample: N = 85. Adjusted R^2^ = 0.21, F_(2, 63)_ = 4.65, p < 0.001*.

The linear regression showed that the best model explaining the severity of borderline symptomatology (*R*${}_{\text{adjusted}}^{2}$ = 0.15, *F* = 4.65, *p* < 0.001) was a model including the number of suicidal attempts realized by the parent (standardized beta = 0.28, *p* = 0.009) and the preoccupied attachment style of the adolescents toward their parents (standardized beta = 0.21, *p* = 0.043) (Table 2).

## Discussion

This study sought to determine whether parental suicidal attempts (and single vs. multiple attempts) could differ in a BPD adolescent population vs. control. First, our study shows that parents of BPD adolescents (specially mothers) made more suicidal attempts than controls. These suicidal attempts happened several years earlier, when patients were still children, or in early adolescence.

Second, the severity of borderline symptomatology seemed to correlate with the number of parental suicidal attempts (the higher the number of suicidal attempts, the increased BPD severity in the offspring) and preoccupied attachment style.

Psychopathology is a frequent underlying cause of suicidal attempts. Severe depression, anxiety and substance abuse are commonly found among suicidal attempters (16). Studies who compared single vs. multiple suicidal attempters in adults showed no differences in axis I disorders but found an increased prevalence of borderline personality disorders and higher impulsivity scores in the latter group (16, 17). Therefore, the likelihood of presenting a personality disorder increases with the number of suicidal attempts. Family members of individuals with BPD may be at higher risk of axis II disorders, including BPD (18, 19) via a potential transgenerational transmission of emotional dysregulation from parent to child (creating insecure attachment patterns). This could explain the impact of parental suicidal attempts on BPD severity in the offspring found in our study as well as the trend to present a preoccupied attachment style.

Our study is somewhat limited by the sampling method, size, and cross-sectional design. Indeed, our data comes from adolescents only, who report their perceived patterns of interaction. Yet number of parental suicidal attempts' report is a rather objective data and less sensible to bias.

## Conclusion

Our results highlight the usefulness of assessing parental suicidal behavior when interviewing patients with BPD traits. This pattern of behavior is overrepresented in this population and might correlate with psychopathological severity in the offspring. This could be useful in clinical practice as an early clue to identify patients with potentially severe clinical profiles and parents needing a specific support. Further research is needed to confirm our findings.

## Ethics Statement

This study was approved by the ethics committee of the Hôtel Dieu Hospital in Paris (authorization n**°** 0611259). Results were collected in an anonymous database according to the requirements of the French national committee for private freedoms. All participants, adolescents and parents, signed informed consent after receiving a full description of the study, explanation of its purpose, and information about the confidentiality of the data.

## Author Contributions

All the authors listed in the manuscript have contributed sufficiently to the project to be included as authors. AM participated in data analysis and interpretation and in writing the manuscript. MS, MC, and AP-S initiated and designed the protocol, collected data, participated in data analysis and interpretation and revising the manuscript. VD participated in data analysis and interpretation and revising the manuscript. All authors read and approved the final manuscript.

### Conflict of Interest Statement

The authors declare that the research was conducted in the absence of any commercial or financial relationships that could be construed as a potential conflict of interest.
